# T Lymphocyte Apoptosis in Systemic Lupus Erythematosus Patients

**Published:** 2013-08

**Authors:** Maryam Rastin, Mahmoud Mahmoudi, Mohammadreza Hatef, Maryam Sahebari, Nafiseh Tabasi, Dariush Haghmorad, Reza Nosratabadi, Shahrzad Zamani, Mahdieh Khazaee, Mitra Masoudian

**Affiliations:** 1 Immunology Research Center, BuAli Research Institute, Faculty of Medicine, Mashhad University of Medical Sciences, Mashhad, Iran; 2 Rheumatology Research Center, Imam Reza Hospital, Faculty of Medicine, Mashhad University of Medical Sciences, Mashhad, Iran; 3 Rheumatology Research Center, Ghaem Hospital, Faculty of Medicine, Mashhad University of Medical Sciences, Mashhad, Iran

**Keywords:** Apoptosis, Autoimmune, Gene expression, Systemic lupuserythema- tosus

## Abstract

***Objective(s):*** Apoptosis is a tightly regulated process and plays a crucial role in autoimmune diseases. Because abnormalities in apoptosis are considered to be involved in the pathogenesis of systemic lupus erythematosus (SLE), in present study we studied the apoptosis in T lymphocytes from Iranian SLE patients at protein and gene expression levels for some molecules which are involved in apoptosis pathways.

***Materials and Methods: ***Thirty five SLE patients (23 female, 12 male), and 20 age matched controls (10 female, 10 male) participated in this study. T lymphocytes were isolated from peripheral blood mononuclear cells (PBMCs) using MACS method. Apoptosis rate was studied at protein level by flow cytometer using Annexin V, and at gene expression level using semi-quantitative RT-PCR method for detection of Fas, FasL, Bcl-2, caspase 8, and caspase 9 genes.

***Results: ***The percentage of apoptotic cells in SLE patients was not different in comparison with controls (20.2% ± 1.4 vs 21.1% ± 1.0), but the expression levels of FasL, caspase 8, and caspase 9 genes in all SLE patients and in female patients were significantly lower than controls; 0.45R vs 0.78R for FasL, 0.74R vs 1.0R for caspase 8, and 0.76R vs 1.26R for caspase 9 in all SLE patients and 0.37R vs 0.82R for FasL, 0.45R vs 1.6R for caspase 8, and 0.63R vs 1.56R for caspase 9 in female patients.

***Conclusion:*** The expression levels of FasL, caspase 8 and caspase 9 molecules involved in apoptosis decreased in female, but not in male SLE patients.

## Introduction

Systemic Lupus Erythematosus (SLE) is a multifactorial autoimmune disease, in which tolerance is loosed and autoantibodies developed against self antigens (1). In normal individuals in the process of tolerance autoreactive T and B cells are deleted (2), but in patients with SLE autoreactive cells persisted and a variety of autoantibodies are generated (3). To understand the pathogenesis of SLE it is important to know how self antigens become available and immunogenic to immune system, many researchers believed that apoptosis play a crucial role in autoimmunity, including SLE (4-6). 

Apoptosis is a tightly regulated process and is essential for cell elimination, lymphocyte appropriate repertoire development and immune response regulation. Disturbances in apoptosis and any defect in clearance of apoptotic cells, increases exposure of modified autoantigens to the immune system (2, 7).

Previous studies on SLE patients revealed enhanced apoptosis rate without any abnormalities in apoptosis machinery (7, 8), but most of these investigations have been performed at protein level, by detecting Annexin V as an early marker for apoptosis (9), or by detecting Fas (CD95) and/or FasL expression using flow cytometry. Both of these methods detects molecules at protein level in surface of apoptotic cells, but accelerated apoptosis or decreased clearance of apoptotic cells could enhance these molecules.

In present research besides studying apoptosis rate at protein level we also intended to study some molecules involved in apoptosis pathways in gene expression levels using semi-quantitative RT-PCR method. As there are two main pathways involved in apoptosis; the extrinsic pathway is activated via ligation of Fas with its ligand "FasL", and the intrinsic pathway is mediated through mitochondria (9, 10), therefore we studied Fas, FasL, Bcl-2, caspase 8, and caspase 9 molecules which are involved in these two pathways.

## Materials and Methods

***Patients and Controls***

The study was carried out in 35 patients with systemic lupus erythematosus; 23 female patients with mean ages of 28 years (16-43 years), 12 male patients with mean ages of 30 years (19-47 years), 20 age matched normal controls; 10 female controls with mean ages of 29 years (25-38 years) and 10 male controls with mean ages of 30 years (25-40 years). All SLE patients met at least 4 ACR (American College of Rheumatism) criteria of SLE. The famale participants in this study were all in mid menstrual cycle (days 5-14), and had regular menstrual cycle, and none of them were taking birth control drugs.

SLE patients were not taking any treatments or taking less than 10 mg/day prednisolone, and/or 200 mg/day hydroxychloroquine, and none of them were on any cytotoxic drugs.

This study was approved by Mashhad University of Medical Sciences Ethics Committee. Written informed consent was obtained from all participating patients and controls. 

***Cell isolation***

Peripheral blood mononuclear cells from lupus erythematosus patients and healthy controls were isolated using Ficoll density gradient centrifugation (GIBCO, USA). Cells were first centrifuged 20 min at 160 g and after removal of 20 ml of supernatant to eliminate platelets, were recentifuged for 20 min at 350 g. Cells in interface layer were transferred to a fresh tube washed in PBS, and then resuspended in 200 l PBS.

***T cell isolation using MACS method***

T lymphocytes were isolated from PBMCs by MACS (Magnetic Cell Sorting) method, using "T Cell Negative Isolation Kit" (Dynal, Norway) which contains depletion beads and a mouse antibody mix for CD14, CD16, CD56, HLA class II DR/DP and CD235a.

The isolated PBMCs were adjusted to 110^5^ cells/ μl (10^7^ cells in total volume of 100 μl), then 20 μl heat inactivated fetal calf serum (FCS) and 20 μl antibody mix were added and incubated 10 min at 4°C. Cells were washed by adding 1 ml of PBS containing 0.1% BSA and centrifuged for 8 min at 500 g. Supernatant removed and cells were resuspended in 0.9 ml PBS containing 0.1% BSA. 100 μl prewashed Dynabeads was added and incubated for 15 min at 20°C with gentle rotation. Cells were pipetted 5-6 times to resuspend probable rosettes. 1 ml PBS containing 0.1% BSA was added and the tube was placed in magnetic device for 2 min. Supernatant which contains negatively isolated T cells was transferred to a fresh tube. More than 95% of isolated cells expressed the CD3 marker when tested by Flow Cytomtry (BD, FACS Calibur).

 One part of isolated T cells was used for diagnosis of apoptosis by Annexin V detection kit and one part was used for RNA isolation, cDNA synthesis and semi-quantitative RT-PCR.

***Apoptosis detection by flow cytometry***

The "Phosphatidyl Serine Detection" kit (IQ Products, Netherland) was used to measure apoptosis of isolated T lymphocytes according to manufacturer´s instructions; one part of isolated T lymphocytes were washed in calcium buffer and adjusted to 1.510^6^ cells/μl . To 100 μl of cells 10 μl FITC labeled Annexin V was added and incubated 20 min on ice in dark, after incubation 2 ml cold calcium buffer was added and cells were washed and centrifuged for 5 min in 300 g. Ten μl propidium iodide was added and incubated 10 min on ice and analyzed by BD flow cytometry. Using the kit we differentiated viable cells (Annexin V^-^ PI^-^) from early apoptotic (Annexin V^+ ^PI^-^), late apoptotic (Annexin V^+^ PI^+^), and necrotic cells (Annexin V^-^ PI^+^).

***RNA isolation***

Isolated T lymphocytes were transferred to a tube, centrifuged and pellet was washed twice in PBS, and total RNA was isolated from these cells using Tripure (Roche, Germany), according to manufacturer´s instructions. RNA was suspended in DEPC treated H2O, and treated with 1 unit/μl DNasI (Fermentas) at room temperature (RT) for 1 hr to exclude DNA.

Extracted RNAs were quantified spectrophotome-trically, and run in agarose gel to determine the RNA quality.

***Semi-quantitative RT-PCR analyses***

cDNAs were synthesized using Random Hexamer primers 0.5 μM (Fermentas), and M-MuLV reverse transcriptase (40 unit) (Fermentas) according to the manufacturer´s instruction. Multiplex-PCR was carried out on Fas, FasL, Bcl-2, caspase 8, caspase 9 as specific genes, and Beta-actin gene as an internal control, using specific primers in a 25 μl reaction in a Biometra Thermal Cycler; 0.4 μM of each specific primers, 0.1 μM of each Beta-actin primers, 200 μM dNTPs (Roche, Germany) and 1 unit Taq DNA polymerase (Roche, Germany). Characteristics of the primers used in this study are listed in Table 1.

PCR products were electrophoresed in 2% agarose gel containing ethidium bromide, and photographed by Imago gel documentation, then analyzed semi-quantitatively using Labworks software.

**Table 1:** Primers used in this study

Primer name	Sequence
FaF	5- AGCTTGGTCTAGAGTGAAAA-3
FaR	5- GAGGCAGAATCAGAGATAT- 3
FLF	5- TCTCAGACGTTTTTCGGCTT-3
FLR	5- AAGACAGTCCCCCTTGAGGT-3
BCF	5- CTGGTGGGAGCTTGCATCAC-3
BCR	5- ACAGCCTGCAGCTTTGTTTC-3
Cs8F	5-TCTGGAGCATCTGCTGTCTG -3
Cs8R	5-CCTGCCTGGTGTCTGAAGTT -3
Cs9F	5-CGTGGTGGTCATTCTCTCTCA -3
Cs9R	5- GTCACTGGGTGTGGGCAAACT-3

***Statistical analysis***

Data is presented as Mean ± SEM and analyzed using SPSS software. Student's t-test was done for

comparisons between SLE patients and control groups. Statistically significant differences were accepted at a *P*<0.05.

## Results

***Apoptosis of T cells in SLE patients***

**Figure 1:**
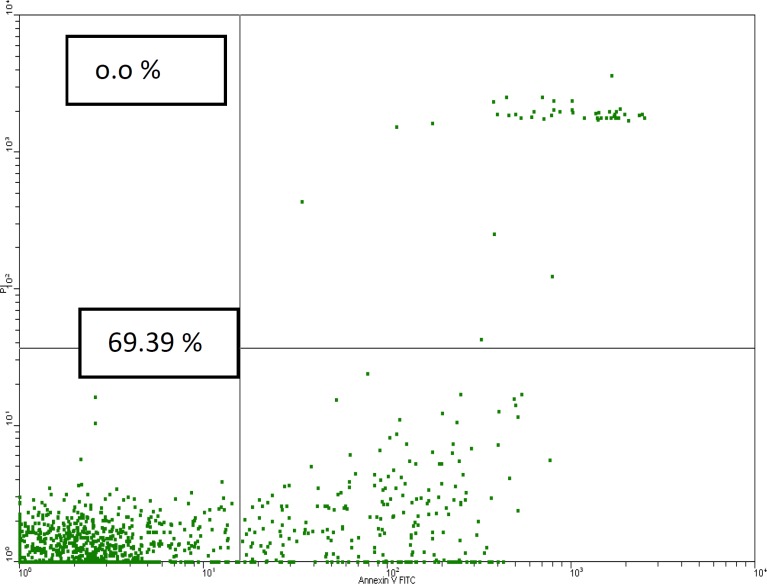
Apoptosis detection by using phosphatidyl Serine Detection kit: viable cells (lower left), early apoptotic cells (lower right), late apoptotic cells (upper right), and necrotic cells (upper left

Using Annexin V binding as an indicator of phosphatidyl serine surface exposure in early apoptotic cells and PI staining as necrosis indicator (Figure 1), we showed that in SLE patients the percentage of early apoptotic cells was not different in comparison with control groups (20.2% ± 1.4 versus 21.1% ± 1.0). In female and male SLE patients the rate of apoptotic cells was also not different in comparison with female and male controls (18.1% ± 1.6 versus 20.6% ± 1.6 for female patients and controls, and 23.5% ± 2.4 versus 19.9% ± 1.0 for male patients and controls respectively).

**Figure 2:**
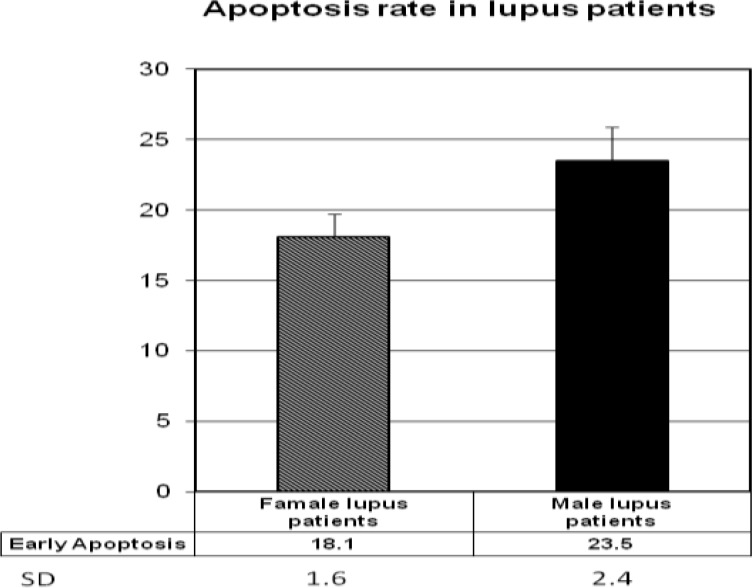
Rate of apoptosis in female and male lupus patients. The percentage of apoptotic cells in male patients is significantly higher than female patients (*P* 0.05

In comparison to female and male SLE patients it was concluded that the percentage of apoptotic cells in male patients is significantly higher than female patients (23.5% ± 2.4 versus 18.1% ± 1.6) (*P*< 0.05) (Figure 2).

***Results of semi-quantitative RT-PCR in SLE patients***

**Figure 3:**
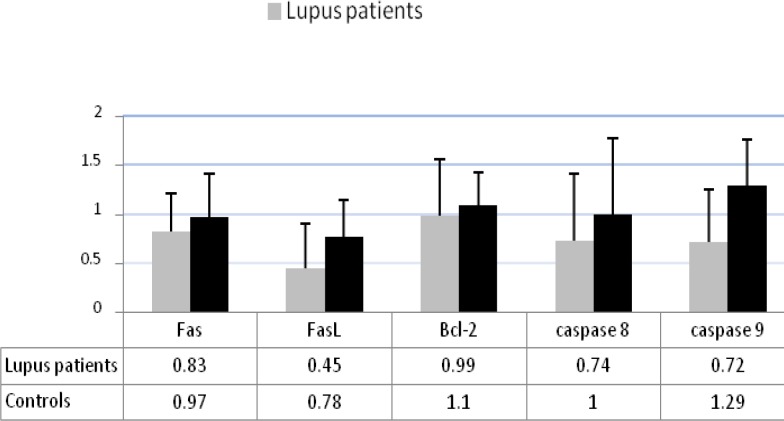
The expression levels of the apoptotic related molecules in lupus patients and in control groups. In lupus patients the expression levels of FasL, caspase 8, and caspase 9 were significantly lower than control groups (*P*<0.05), but there was no differences in the expression levels of Fas and Bcl-2 between two groups

The expression level of Fas, FasL, Bcl-2, caspase 8, and caspase 9 molecules involved in apoptosis machinery in SLE patients and control group was investigated. The present results showed that in SLE patients the expression levels of FasL, caspase 8, and caspase 9 were significantly lower than control groups (0.45R versus o.78R for FasL, 0.74R versus 1.0R for caspase 8, and 0.76R versus 1.26R for caspase 9) (*P* <0.05), but the expression level of Fas and Bcl-2 were not significantly different in comparison with control groups (Figure 3). 

***Results of semi-quantitative RT-PCR in female SLE patients***

**Figure 4:**
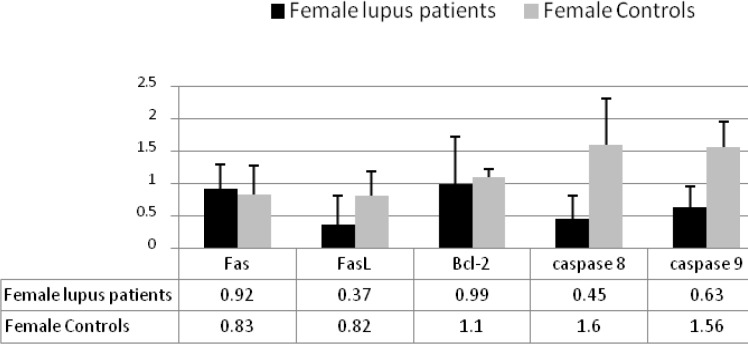
The expression levels of the apoptotic related molecules in female lupus patients and in female controls. The expression levels of FasL, caspase 8, and caspase 9 in female patients were significantly lower than female controls (*P*<0.05), but there was no differences in the expression levels of Fas and Bcl-2 between two groups

To determine the expression levels of molecules involved in apoptosis pathways in female patients we studied 23 female SLE patients and 10 age matched female controls and demonstrated that the expression levels of FasL, caspase 8, and caspase 9 in female patients were significantly lower than female controls (0.37R versus o.82R for FasL, 0.45R versus 1.6R for caspase 8, and 0.63R versus 1.56R for caspase 9) (*P* <0.05), but there were no significant differences in the expression levels of Fas and Bcl-2 between female patients and female controls (Figure 4).

**Figure 5:**
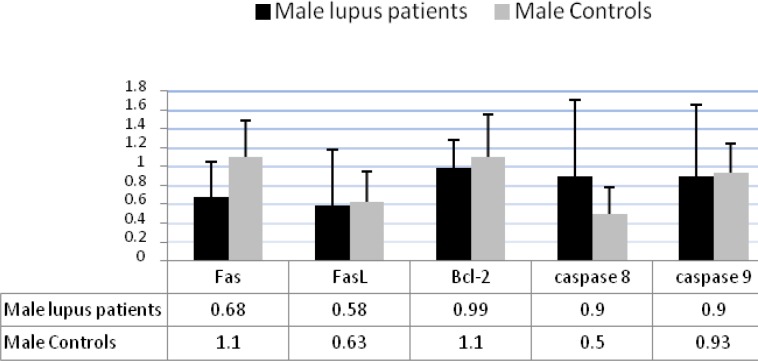
The expression levels of the apoptotic related molecules in male lupus patients and in male controls**. **The expression levels of caspase 8 in male patients was significantly higher than male controls (*P*<0.05), but the expression levels of Fas, FasL, Bcl-2, and caspase 9 was not different in male patients in comparison to male controls

**Figure 6:**
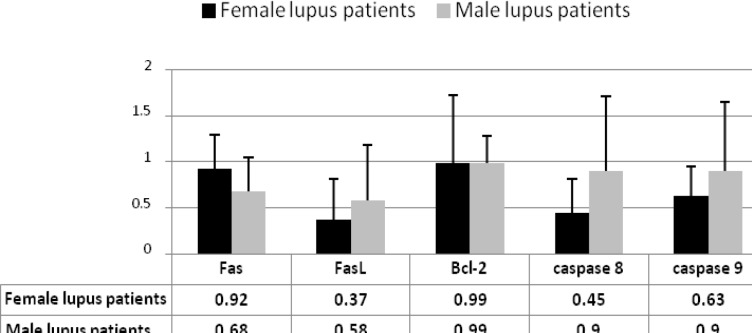
The expression levels of the apoptotic related molecules in female and male lupus patients**. **The expression levels of caspase 8 in female patients was significantly lower than male patients (*P*<0.05), but the expression levels of Fas, FasL, Bcl-2, and caspase 9 was not different between two groups

***Results of semi-quantitative RT-PCR in male SLE patients***

To determine the expression levels of the molecules involved in two different apoptosis pathways in male SLE patients 12 male lupus patients and 10 ages matched male controls were studied. In the present findings the expression levels of caspase 8 in male SLE patients were found to be significantly higher than male controls (0.90R versus 0.50R (*P*<0.05), but there was no significant difference in the expression levels of Fas, FasL, Bcl-2, and caspase 9 in male patients in comparison with male controls (Figure 5).

***Comparison of Results between female and male SLE patients***

The expression levels of FasL, caspase 8, and caspase 9 in female lupus patients were lower than male patients, but only for caspase 8 the difference was significant (0.45R, versus 0.9R for female and male SLE patients) (*P* <0.05), (Figure 6).

## Discussion

Apoptosis is a highly controlled process (11), and plays an important role in pathogenesis of SLE. In current study, we examined apoptosis in gene expression and protein levels using flow cytometry and semi-quantitative RT-PCR. 

The results of this study showed that there was no significant difference in apoptosis rate in protein level neither among lupus patients and control groups nor between male and female patients with their appropriate controls, which was in accordance with results of some previous studies (2, 12). In a number of previous studies, apoptosis rate has been reported to be increased in patients with SLE (4, 5, 13), whereas in other studies no difference was observed between SLE patients and controls when Fas molecule was assessed instead of apoptosis (5, 14, 15), which was consistent with our results. Various factors affect apoptosis both *in vivo* and *in -vitro*, and drug consumption is among these factors. 

Corticosteroids used for treatment of SLE are among the most potent stimulators of apoptosis (16). Most SLE patients enrolled in the present study (23 female and 12 male) were in the early stages of SLE and either took no drugs or consumed a maximum of 10 mg/day corticosteroids and/or 200 mg/day hydroxychloroquine. None of the patients received cytotoxic drugs. Xue *et al *studied 22 patients in inactive stage and 17 patients in the active stage of SLE, and reported that the apoptosis rate was increased in these patients, while some of their patients received more than 10 mg/day corticosteroids (2). Wang *et al *reported increased apoptosis rate in 49 SLE patients, while nothing was mentioned in their study about the medications used for patients (4). Bijl *et al *(8) and Caricchio *et al* (12) studied 13 and 25 SLE patients with similar drug dose as our study, respectively, and reported that the percentage of apoptotic cells and the expression level of Fas molecule was the same in SLE patients and healthy controls, which was comparable to our results. Different study results seem to be to some extent influenced by differences in patients' medication regimens. The differences may also be partly affected by different methodologies used in several studies. 

Results of molecular studies in the present study showed that the expression level of FasL, caspase 8 and caspase 9 genes was decreased in SLE patients and in female, which was in agreement with the results of some previous studies (17). Mass *et al *(2002) assessed the expression level of a number of genes involved in apoptosis in SLE patients, and observed the reduced level of caspase 8 in these patients (18). However, results of some previous studies were different (19), in which apoptotic molecules were examined in protein level, while we investigated the expression of these molecules in gene expression level using semi-quantitative RT-PCR, and differences in procedure may have caused differences in the results. 

In this study, regarding the decline observed in the expression of some apoptosis related genes, we expected the rate of apoptosis in protein level to be decreased in SLE patients, whereas apoptosis rate was not different in patients and controls, perhaps due to various factors including defects in the clearance of apoptotic cells, which could result in accumulation of apoptotic cells. Many studies have reported the defects in the clearance of apoptotic cells in SLE patients (20-22). 

Defects in apoptosis pathway genes and in clearance of apoptotic cells can lead to persistence of autoreactive cells in the developmental phase of the immune system and also when the immune responses subside after elimination of infectious agents.

Comparing the results of male and female SLE patients in present study we showed that apoptosis rate was decreased, and expression of caspase 8 in gene level was lower in female than male (the expression of FasL and caspase9 was also reduced, but was not statistically significant). The reason for this difference between male and female patients is not clear, but differences in sex hormones may be involved (23). Previous studies of our research team (24) and others indicated that the level of prolactin was increased in female and male SLE patients, and the level of DHEA and progesterone as hormones having immunosuppressive effects were decreased (25). 

Future studies in male and female SLE patients will expand our knowledge about the influence of sex hormones on apoptosis in these patients. 

## Conclusion

The results of present study suggest that in female SLE patients there are some defects in apoptosis pathways, while in male patients there is a tendency for enhanced apoptosis and apoptosis related molecules.

## References

[1] Arbuckle MR, McClain MT, Rubertone MV, Scofield RH, Dennis GJ, James JA (2003). Development of auto antibodies before the clinical onset of systemic lupus erythematosus. N Engl J Med.

[2] Xue C, Lan-Lan W, Bei C, Jie C, Wei-Hua F (2006). Abnormal Fas/FasL and caspase-3-mediated apoptotic signaling pathway of T lymphocyte subset in patients with systemic lupus erythematosus. Cell Immunol.

[3] Kaplan MJ (2004). Apoptosis in systemic lupus erythematosus. Clin Immunol.

[4] Wang H, Xu J, Ji X, Yang X, Sun K, Liu X (2005). The abnormal apoptosis of T cell subsets and apoptosis involvement of IL-10 in systemic lupus erythematosus. Cell Immunol.

[5] Munoz L, Bavel CV, Franz S, Berden J, Hermann M, Van der Vlag J (2008). apoptosis in the pathogenesis of systemic lupus erythematosus. Lupus.

[6] Reefman E, Horst G, Nijk MT, Limburg PC, Kallenberg CGM, Bijl M (2007). Opsonization of Late Apoptotic Cells by Systemic Lupus Erythematosus Autoantibodies Inhibits Their Uptake via an Fcγ Receptor–Dependent Mechanism. Arthritis Rheum.

[7] Bijl M, Limburg PC, Kallenberg CG (2001). New insight into the pathogenesis of systemic lupus erythematosus (SLE): the role of apoptosis. N J Med.

[8] Bijl M, Horst G, Limburg PC, Kallenberg CG (2001). Anti-CD3-induced and anti- Fas -induced apoptosis in systemic lupus erythematosus. Clin Exp Immunol.

[9] Zimmermann KC, Bonzon C, Green DR (2001). The machinery of programmed cell death. Pharmacol Ther.

[10] Bengtsson AA, Gullstrand B, Truedsson L, Sturfelt G (2008). SLE serum induces classical caspase-dependent apoptosis independent of death receptors. Clin Immunol.

[11] Krueger A, Fas SC, Baumann S, Krammer PH (2003). The role of CD95 in the regulation of peripheral T-cell apoptosis. Immunol Rev.

[12] Caricchio R, Cohen PL (1999). Spontaneous and induced apoptosis in systemic lupus erythematosus: multiple assays fail to reveal consistent abnormalities. Cell Immunol.

[13] Grondal G, Traustadottir KH, Kristjansdottir H, Lundberg I, Klareskog L, Erlendsson K (2002). Increased lymphocyte apoptosis/ necrosis and IL-10 producing cells in patients and their spouses in Icelandic systemic lupus erythematosus multicase families. Lupus.

[14] Courtney PA, Crockard AD, Williamson K, McConnell J, Kennedy RJ, Bell AL (1999). Lymphocyte apoptosis in systemic lupus erythematosus: relationship with Fas expression, serum soluble Fas and disease activity. Lupus.

[15] Lorenz HM, Grunke M, Hieronymus T, Hermann M, Kuhnel A, Manger B (1997). In vitro apoptosis and expression of apoptosis- related molecules in lymphocytes from patients with systemic lupus erythematosus and other autoimmune diseases. Arthritis Rheum.

[16] Ho CY, Wong CK, Li EK, Lam WK (2001). Effects of dexamethasone on the expression of Fas molecules and apoptosis of lymphocytes in patients with systemic lupus erythematosus. Immunol Invest.

[17] McNally J, Yoo DH, Drappa J, Chu JL, Yagita H, Friedman SM (1997). Fas ligand expression and function in systemic lupus erythematosus. J Immunol.

[18] Maas K, Chan S, Parker J, Slater A, Moore J, Olsen N (2002). Cutting edge; molecular portrait of human autoimmune disease. J Immunol.

[19] Kovacs B, Liossis SN, Dennis GJ, Tsokos GC (1997). Increased expression of functional Fas-ligand in activated T cells from patients with systemic lupus erythematosus. Autoimmunity.

[20] Gaipl US, Munoz LE, Grossmayer G, Lauber K, Franz S, Sarter K (2007). Clearance deficiency and systemic lupus erythematosus (SLE). J Autoimmun.

[21] Grossmayer GE, Munoz LE, Gaipl US, Franz S, Sheriff A, Voll RE (2005). Removal of dying cells and systemic lupus erythematosus. Mod Rheumatol.

[22] Shao WH, Cohen PL (2011). Disturbances of apoptotic cell clearance in systemic lupus erythematosus. Arthritis Res Ther.

[23] Rastin M, Hatef MR, Tabasi N, Mahmoudi M (2012). The pathway of estradiol- induced apoptosis in patients with systemic lupus erythematosus. Clin Rheumatol.

[24] Rastin M, Hatef MR, Tabasi N, Sheikh A, Morad Abbasi J, Mahmoudi M (2007). Sex hormones and peripheral white blood cells in systemic lupus erythematosus. Iran J Immunol.

[25] Zandman-Goddard G, Peeva E, Shoenfeld Y (2007). Gender and autoimmunity. Autoimmun Rev.

